# The prevalence of depression in axial spondyloarthritis and its association with disease activity: a systematic review and meta-analysis

**DOI:** 10.1186/s13075-018-1644-6

**Published:** 2018-07-11

**Authors:** Sizheng Zhao, Daniel Thong, Natasha Miller, Stephen J. Duffield, David M. Hughes, Laura Chadwick, Nicola J. Goodson

**Affiliations:** 1 Musculoskeletal biology I, Institute of Ageing and Chronic Disease, University of Liverpool, Liverpool, UK; 2 Department of Academic Rheumatology, Aintree University Hospital, Liverpool, UK; 3 Department of Biostatistics, Institute of Translational Medicine, University of Liverpool, Liverpool, UK

**Keywords:** Axial spondyloarthritis, Ankylosing spondylitis, Depression, Prevalence, Mental health, Comorbidity, Meta-analysis, Disease activity, Functional impairment

## Abstract

**Background:**

Depression is common among patients with axial spondyloarthritis (axSpA), but reports of its prevalence are highly variable. We performed a systematic review to (i) describe the prevalence of depression in axSpA, (ii) compare its prevalence between axSpA, ankylosing spondylitis (AS) and non-radiographic axSpA (nr-axSpA) cohorts, and (iii) compare disease activity and functional impairment between those with and without depression.

**Methods:**

We searched Medline, PubMed, Web of Science, PsycINFO, CINAHL Plus, the Cochrane library and conference abstracts of the European League Against Rheumatism, British Society for Rheumatology and American College of Rheumatology using a predefined protocol in accordance with Preferred Reporting Items for Systematic reviews and Meta-Analyses (PRISMA) guidelines. Meta-analysis was performed using quality-effects model.

**Results:**

Fifteen original articles and one abstract were included for analysis; 14 studies described AS cohorts and two nr-axSpA. Three screening criteria and one diagnostic criterion were used to define depression. Prevalence ranged from 11 to 64% depending on criteria and thresholds used. Pooled prevalence of at least moderate depression was 15% using the Hospital Anxiety and Depression Scale (HADS) threshold of ≥ 11. The prevalence of depression was similar between axSpA, AS and nr-axSpA cohorts. Patients with depression had significantly worse disease activity, including higher BASDAI by 1.4 units (95% CI 1.0 to 1.9), ASDAS by 0.5 units (95% CI 0.3 to 0.7) and ESR by 3.5 mm/h (95% CI 0.6 to 6.4). They also had greater functional impairment with higher BASFI and BASMI by 1.2 units (95% CI 0.6 to 1.8) and 0.6 units (95% CI 0.3 to 0.8), respectively. Mean age of each study cohort inversely correlated with depression prevalence.

**Conclusions:**

Depression is common among axSpA patients and is associated with more severe disease activity and functional impairment. Identifying and managing depression should form part of their holistic care. Further longitudinal studies are needed to explore the impact of depression on treatment outcomes and axSpA treatment on symptoms of depression.

**Electronic supplementary material:**

The online version of this article (10.1186/s13075-018-1644-6) contains supplementary material, which is available to authorized users.

## Background

Axial spondyloarthritis (axSpA) is a chronic inflammatory disease which predominantly affects the axial skeleton. It can be divided into ‘radiographic’ (ankylosing spondylitis, AS) and ‘non-radiographic’ (nr-axSpA), depending on whether definitive structural changes are evident on plain radiographs of sacroiliac joints [1, 2]. They are both characterised by inflammatory pain and functional impairment [3].

Like other conditions for which chronic pain is a feature, axSpA is known to be associated with depression [4, 5]. However, quantifying the prevalence of depression is challenging. Formal diagnosis of depression requires expertise and time consuming assessments. Instead, screening questionnaires are often used in both clinical and research settings. There are many different screening tools, each with variable score thresholds to define depression.

Estimating the prevalence of depression in axSpA is further complicated by the distinction of AS and nr-axSpA. Although they can be considered entities along a spectrum, there are key differences: the proportion of male patients is higher in AS than nr-axSpA cohorts, functional impairment is greater in AS cohorts, and AS is more commonly associated with elevated inflammatory markers [6]. Gender, functional impairment and inflammation can all potentially influence the likelihood of developing depression [7–9].

In spite of these challenges, quantifying the prevalence of depression is an important first-step to improve awareness and management of this comorbidity. Depression has direct importance to rheumatologists since current methods of disease severity assessments, on which important treatment decisions are made, rely on subjective patient-reported measures, such as the Bath AS Disease Activity Index (BASDAI) and spinal pain visual analogue scale (spVAS). The presence of depression is known to influence the reporting of pain and impairment [5]. Equally, more severe disease likely increases the risk of developing depression. It has been shown that self-reported anxiety and depression in axSpA have greater associations with disease activity and functional impairment than smoking or deprivation [10].

To date, depression in axSpA has not been systematically reviewed. Our aims were therefore to (i) report pooled prevalence of depression in axSpA, (ii) compare the prevalence of depression between axSpA, AS and nr-axSpA groups, and (iii) compare disease activity and functional impairment between those with and without comorbid depression.

## Patients and methods

A systematic review was performed in accordance with the Preferred Reporting Items for Systematic Reviews and Meta-Analyses (PRISMA) guidelines [11]. The protocol for this review was pre-registered in advance (PROSPERO: CRD42017082359). Two reviewers (SZ, DT) searched Medline, PubMed, Web of Science, PsycINFO, CINAHL plus and the Cochrane Library for relevant literature in February 2018. In addition, abstract archives of the European League Against Rheumatism (EULAR), British Society for Rheumatology (BSR) and American College of Rheumatology (ACR) were searched up to, and including, 2017. The following search term was used: depress* AND (ankylosing OR spondyloarthr*). Differential nomenclature for depression (affective disorder, mood disorder, adjustment disorder, dysthymia) did not affect results in preliminary searches.

Studies were included if they: (i) were cross-sectional in design or were longitudinal studies reporting baseline data, (ii) used a validated diagnostic or screening criteria for depression with a defined cut-off threshold, and (iii) recruited adult patients fulfilling the modified New York or ASAS criteria for AS or axSpA, respectively. Studies were excluded if they used non-representative sampling (highly selective recruitment criteria, for example, studies that only recruited women) or had a samples size of less than 30 (to avoid unreliable prevalence estimates). Publications in abstract form only were also considered, as some prevalence studies may not be published as full articles and may have sufficiently described methodology within the restrictions of an abstract. Reviews, comments and editorials were excluded.

Two reviewers (DT, NM) independently assessed study eligibility and extracted data from qualifying studies. Any discrepancies were resolved through discussion moderated by a third reviewer. Information from included studies was extracted into predefined tabulated summaries containing data on: depression criteria and threshold used, study design, sample size, country setting, mean age, percentage of males, and depression prevalence.

Studies were assessed for risk of bias (SZ, NM) using the Health States Quality Index (Additional file 1: Table S1). This is an 11-point scoring system that assesses studies under the following headings: target population and observation period, diagnostic criteria, case ascertainment, measurement administration, catchment area and prevalence measure [12]. For purposes of the below meta-analysis, the score was divided by 10 to derive a quality parameter with an upper limit of 1.

### Statistical analysis

Prevalence estimates were reported as percentages (95% confidence interval (CI), I^2^ statistic). When performing meta-analysis of proportions (variable bound between 0 and 1), traditional weighting methods based on inverse variance are problematic when the proportions are close to the bound limits, giving more weight to such studies [13]. The double arcsine transformation was therefore used. Results were pooled according to depression criteria and threshold using quality-effects models. This method redistributes weights by accounting for the above quality parameter and can be considered as an extension of the random-effects model. Sub-group meta-analyses by diagnostic category were also performed. All estimates were also presented using random-effects models (DerSimonian-Laird). Heterogeneity of meta-analysis estimates were presented using the I^2^ statistic. Funnel plot and the Doi plot/LFK index [13] were used to assess risk of publication bias.

Commonly used thresholds for each screening tool were used to categorise severity of depression. For the Hospital Anxiety and Depression Scale depression subscale (HADS) ≤ 7 was interpreted as no depression, 8 to 10 mild, 11 to 14 moderate and ≥ 15 severe [14]. For the Zung Self-Rating Depression Scale (SDS) ≤ 49 was interpreted as no depression, 50 to 69 depression, ≥ 70 severe depression [15]. The Patient Health Questionnaire (PHQ-9) threshold for depression has been suggested to be between 8 and 11, depending on context [16]. Studies that used similar thresholds were grouped together. If a study reported depression prevalence using two different thresholds, it could contribute to more than one pooled estimate. Sensitivity analyses were performed: studies reporting outlying prevalence estimates were critically reviewed and, if appropriate, excluded to evaluate changes in prevalence and heterogeneity. The association between study characteristics and reported depression prevalence was assessed using Spearman’s rank correlation (r_s_).

Differences in markers of disease activity and functional impairment were compared between groups with and without depression. Weighted mean difference (WMD) was calculated for each marker of disease activity and functional impairment, with pooled estimates shown in forest plots. Analyses were performed using MetaXL version 5.3 (Sunrise Beach, Australia; http://www.epigear.com/).

## Results

A total of 769 full-text publications and 40 conference abstracts were found from the literature search. These titles and abstracts were screened for eligibility. After excluding duplicates and irrelevant studies, 70 studies were assessed for full-text eligibility, from which 15 full-text articles [17–31] and one abstract [32] were included in the analysis. Among the excluded studies, two [33, 34] used the same cohort as the study by Healey et al. [20] and two [35, 36] reported the same cohort as Hakkou et al. [26]. A summary of the selection process is shown in the flowchart (Additional file 1: Figure S1).

Of the 16 included studies, 14 recruited participants with AS (including three axSpA studies which reported on AS participants separately) and two reported on nr-axSpA cohorts (including one axSpA study which reported on nr-axSpA participants separately). Sample size ranged from 60 to 1504. A total of 4753 axSpA patients were included across all studies, including separately described groups of 2857 AS and 334 nr-axSpA patients. Twelve studies were cross-sectional in design, three were longitudinal, and one was a randomised controlled trial (RCT). Four studies were from Turkey, five from China (including one from Hong Kong), three from the UK, and one each from Greece, Morocco and Spain (Canary Islands). The RCT recruited from Europe, Asia and South America.

Three screening criteria and one diagnostic criterion were used for identifying depression. Nine studies used HADS with three different thresholds, five used SDS with three different thresholds, and one used PHQ-9. For the purposes of meta-analyses, HADS ≥ 7/8 were grouped together, and SDS ≥ 50/51/53 were grouped together. Only one study used diagnostic criteria for depression, the Structured Clinical Interview for Diagnostic and Statistical Manual of Mental Disorders (SCID).

Given the strict inclusion criteria employed for this meta-analysis, most studies had high quality scores with one scoring 6, three scoring 8, nine scoring 9 and three scoring ≥ 10 on the Health States Quality Index (Additional file 1: Table S2).

### Prevalence of depression

The prevalence of depression ranged from 11 to 64%, depending on criteria and threshold used to identifying disease. Table 1 summarises the study characteristics, depression prevalence and quality score. Funnel/Doi plots and the LFK index suggested no evidence of publication bias (Additional file 1: Figure S2).

**Table 1:** Summary of study characteristics, prevalence of depression and quality of studies included in this meta-analysis

Depression criteria (threshold)	Study	Study design	Diagnosis	Sample size	Country	Age, mean (SD)	Males, %	Depression prevalence, %	Quality index
HADS (≥ 7)	Baysal 2011 [27]	Cross-sectional	AS	243	Turkey	34.7 (10.4)	86.4	39.5	0.8
	Kilic 2014 [18]	Cross-sectional	axSpA	316	Turkey	36.3 (9.5)	63.0	44.0	0.9
			AS	174		38.3 (NS)	NS	45.4	
			nr-axSpA	142		33.9 (NS)	NS	42.3	
HADS (≥ 8)	Healey 2011 [20]	Cross-sectional	AS	612	UK	50.8 (12.2)	71.6	32.0	1.0
	Hakkou (a) 2011 [26]	Cross-sectional	AS	110	Morocco	38.5 (12.6)	68.2	55.5	0.9
	Ates 2015 [32]	Cross-sectional	AS	60	Turkey	NS	66.7	43.3	0.7
	Dougados (a) 2017 [25]	RCT	nr-axSpA	192	Europe, Asia, and South America	31.9 (7.8)	60.1	32.3	0.9
HADS (≥ 11)	Martindale 2006 [21]	Longitudinal	AS	89	UK	Median (IQR): 50 (38.5–55.5)	83.1	12.4	0.9
	MacFarlane 2017 [30]	Longitudinal	axSpA	1504	UK	Median (IQR): 51.2 (40.1–63.1)	68	13.8	1.0
	Hakkou (b) 2011 [26]	Cross-sectional	AS	110	Morocco	38.5 (12.6)	68.2	37.3	0.9
	Rodríguez-Lozano 2012 [29]	Cross-sectional	AS	190	Spain	48.4 (11.7)	75.3	10.5	0.9
	Dougados (b) 2017 [25]	Cross-sectional	nr-axSpA	192	Europe, Asia, and South America	31.9 (7.8)	60.1	15.6	0.9
SDS (≥ 50)	Günaydin 2009 [23]	Cross-sectional	AS	62	Turkey	39.6 (10.3)	83.9	27.4	0.9
	Jiang 2018 [31]	Cross-sectional	AS	683	China	27.3 (8.7)	80.4	64.0	1.0
SDS (≥ 51)	Zhang 2016 [17]	Cross-sectional	AS	314	China	27.6 (8.3)	74.5	35.4	0.8
SDS (≥ 53)	Xu 2016 [22]	Cross-sectional	AS	103	China	32.9 (10.7)	75.7	36.9	0.8
	Zou 2016 [28]	Cross-sectional	axSpA	60	China	31.8 (10.1)	73.3	43.3	0.8
			AS	40		31.5 (10.1)	70.0	42.5	
PHQ-9 (≥ 10)	Hyphantis 2013 [19]	Longitudinal	AS	55	Greece	42.9 (10.9)	85.5	14.5	0.9
SCID	Chan 2017 [24]	Cross-sectional	axSpA	160	Hong Kong	46.2 (12.7)	70.6	10.6	0.9
			AS	122		NS	NS	11.5	

*HADS* Hospital Anxiety and Depression Scale—depression subscale, *SDS* Zung self-rating depression scale, *PHQ9* Patient Health Questionnaire, *SCID* Structured Clinical Interview for Diagnostic and Statistical Manual of Mental Disorders, *AS* ankylosing spondylitis, *axSpA* axial spondyloarthritis, *nr-axSpA* non-radiographic axSpA, *NS* not specified, *IQR* interquartile range

**Fig. 1:**
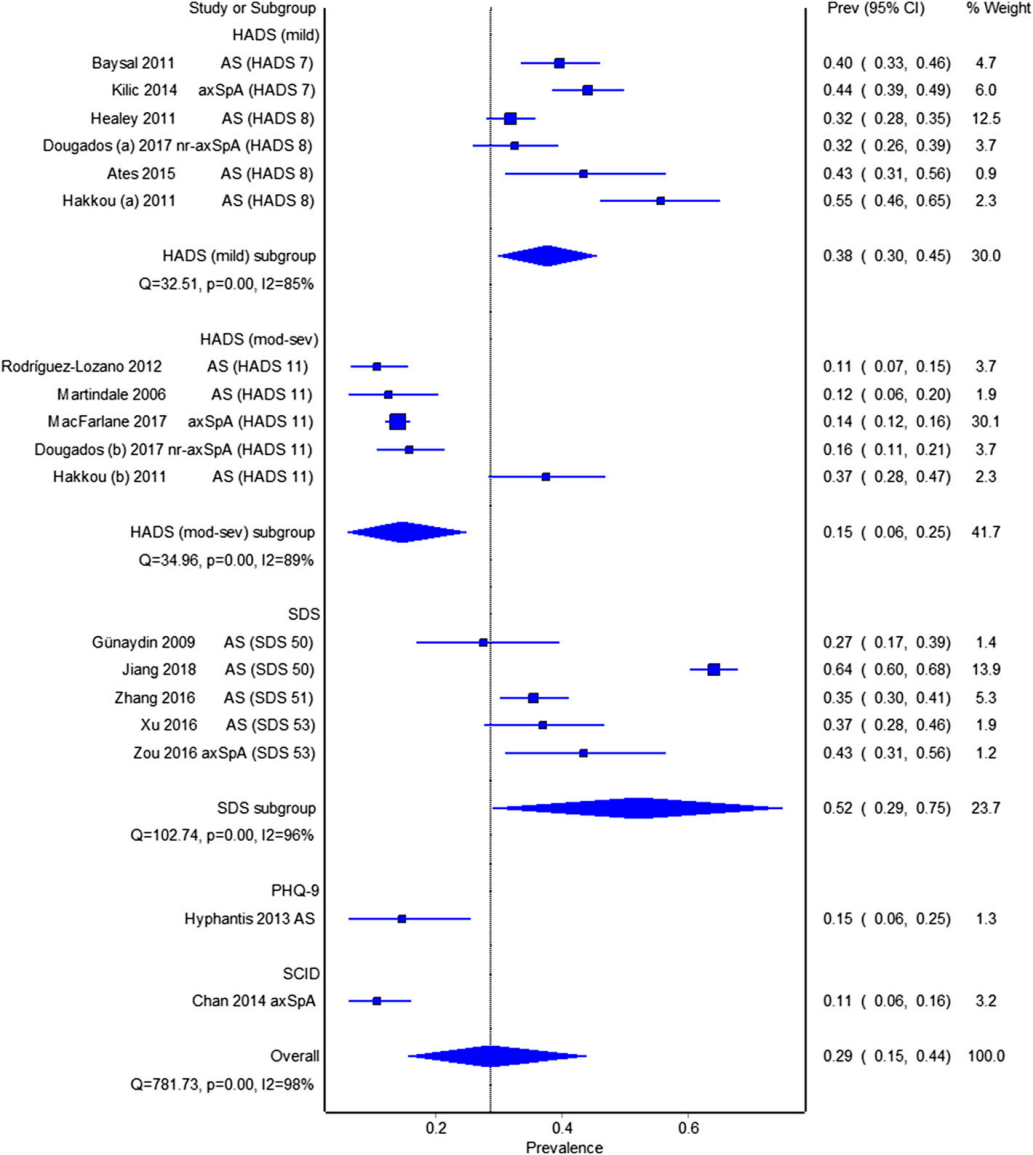
Pooled prevalence of depression in axSpA cohorts, grouped by criteria and threshold

Figure 1 shows a forest plot of prevalence estimates using quality-effects models, grouped by criteria and threshold. Pooled prevalence of mild depression (HADS ≥ 7/8) was 38% (95% CI 30 to 45%, I^2^ = 85%). Pooled prevalence of at least moderate depression, using HADS ≥ 11, was 15% (95% CI 6 to 25%, I^2^ = 89%) and using SDS was 52% (95% CI 29 to 75%, I^2^ = 96%). The study by Hyphantis et al. [19] reported 15% depression using PHQ9 ≥ 10. The study by Chan et al. [24] reported a prevalence of 11% for major depressive disorder using the SCID reported. Pooled prevalence using both quality- and random-effects meta-analysis are shown in Table 2.

**Table 2:** Summaries of pooled depression prevalence grouped by screening criteria and thresholds used

		Quality-effects model		Random-effects model		Sensitivity analysis	
		Pooled prevalence	95% CI, I^2^	Pooled prevalence	95% CI, I^2^	Pooled prevalence	95% CI, I^2^
All studies	HADS (≥ 7/8)	38%	30 to 45%, I^2^ = 85%	40%	47 to 33%, I^2^ = 85%	36%	30 to 42%, I^2^ = 77%
	HADS (≥ 11)	15%	6 to 25%, I^2^ = 89%	17%	11 to 24%, I^2^ = 89%	14%	12 to 15%, I^2^ = 0%
	SDS	52%	29 to 75%, I^2^ = 96%	41%	26 to 58%, I^2^ = 96%	36%	31 to 40%, I^2^ = 13%
AS	HADS (≥ 7/8)	38%	28 to 48%, I^2^ = 86%	38%	28 to 48%, I^2^ = 86%	36%	28 to 45%, I^2^ = 79%
	HADS (≥ 11)	18%	3 to 36%, I^2^ = 94%	18%	3 to 36%, I^2^ = 94%	11%	8 to 15%, I^2^ = 0%
	SDS	52%	28 to 76%, I^2^ = 96%	41%	25 to 58%, I^2^ = 96%	35%	31 to 39%, I^2^ = 0%
nr-axSpA	HADS (≥ 7/8)	36%	27 to 46%, I^2^ = 71%	36%	27 to 46%, I^2^ = 71%	NA	NA

Estimates were presented by quality-effects model, random-effects model, and sensitivity analysis excluding the studies by Hakkou et al. and Jiang et al.

*HADS* Hospital Anxiety and Depression Scale—depression subscale, *SDS* Zung self-rating depression scale, *AS* ankylosing spondylitis, *axSpA* axial spondyloarthritis, *nr-axSpA* non-radiographic axSpA, *NA* not applicable

Two studies reported disproportionately high prevalence of depression. Hakkou et al. [26] attributed this to the cohort’s low socioeconomic status. Excluding this study improved the HADS subgroup heterogeneity without altering the pooled estimates significantly (Table 2). The Chinese study by Jiang et al. [31] reported the highest depression prevalence (64%). This cohort had the lowest mean age (27 years) and reported a low participation rate, with only 25% (683/2772) of the total cohort completing the required assessments. Excluding this study reduced both heterogeneity and prevalence estimate (52 to 36%) for the SDS group.

Prevalence of depression was inversely associated with age (r_s_ = − 0.71, *P* = 0.003) but not with study size, BASDAI, year of publication or proportion of males (data not shown).

### Comparing axSpA, AS and nr-axSpA cohorts

Fourteen of 16 included studies reported the prevalence of depression for AS cohorts. The pooled prevalences for AS cohorts are shown in Table 2. Again, the studies by Hakkou et al. and Jiang et al. reported high prevalence of depression (Additional file 1: Figure S3) and were excluded in sensitivity analyses.

The studies by Chan et al. [24], Zou et al. [28] and Kilic et al. [18] reported similar prevalence of depression between axSpA and their AS subgroups (Table 1). Kilic et al. also reported similar prevalence between AS and nr-axSpA subgroups (45.4 vs 42.3%, *P* = 0.58).

Two studies reported depression prevalence for nr-axSpA cohorts. Pooled prevalence of mild depression (HADS ≥ 7/8) for nr-axSpA was similar to that of AS cohorts (Table 2).

### Comparing markers of disease severity between groups with and without depression

**Fig. 2:**
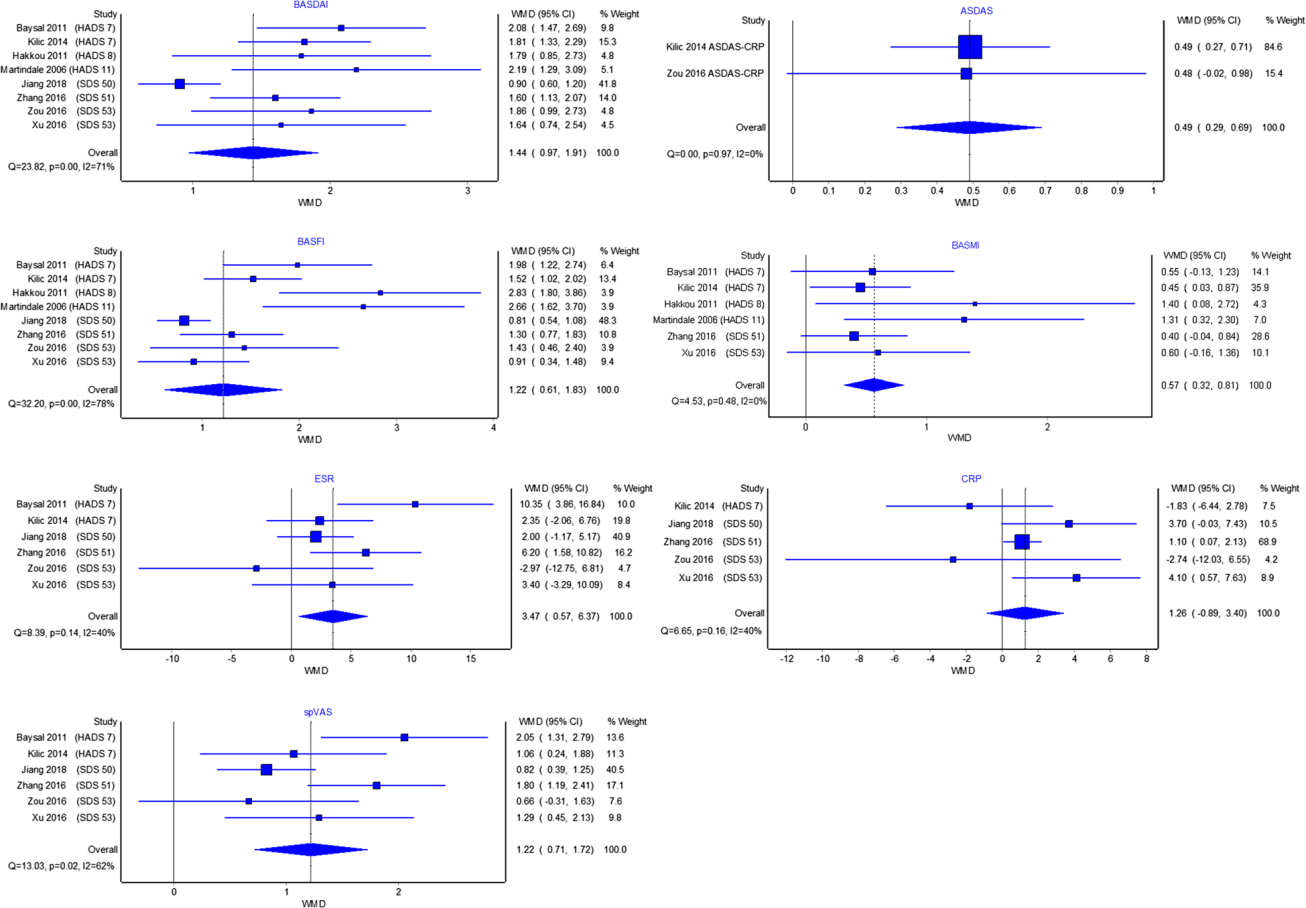
Measures of disease activity and functional impairment are worse in axial spondyloarthritis patients with comorbid depression. Effect sizes shown as weighted mean difference (*WMD*)

Eight studies compared markers of disease severity between groups with and without depression (Additional file 1: Table S3). For BASDAI, spVAS and BASFI, most studies reported significantly higher scores in the group with depression compared to those without, regardless of criteria or threshold used to define depression (Fig. 2). Across the depressed groups, scores were generally worse for the Bath AS metrology index (BASMI), AS disease activity score (ASDAS), CRP and ESR, but few individual comparisons were statistically significant.

All eight studies reported significantly worse BASDAI in the group with depression. Despite the variety of criteria and thresholds used, the weighted mean differences (WMDs) were similar. Pooling WMDs, BASDAI was 1.4 units (95% CI 1.0 to 1.9) higher in the depressed group. Of the six studies that reported spVAS, the groups with depression scored 1.2 units (95% CI 0.7 to 1.7) higher. Only two studies reported ASDAS, with a pooled WMD of 0.5 units (95% CI 0.3 to 0.7) between the two groups. ESR (3.5 mm/h, 95% CI 0.6 to 6.4 mm/h) was significantly higher in groups with depression, but not CRP (1.3 mg/dl, 95% CI − 0.9 to 3.4).

All studies reported significantly worse BASFI in the group with depression. The pooled difference in BASFI was 1.2 units (95% CI 0.6 to 1.8) but with more variation among the studies. The group with depression in the study by Hakkou et al. had much poorer function (BASFI, BASMI) compared to depressed groups of other studies, despite using a threshold for ‘mild’ depression. In contrast to other Bath indices, not all studies reported a difference in BASMI when comparing groups with and without depression. Nevertheless, the pooled estimate showed that axSpA patients with comorbid depression had significantly higher BASMI than those without (0.6 units, 95% CI 0.3 to 0.8).

## Discussion

This systematic review and meta-analysis of nearly five thousand axSpA patients has shown that depression is highly prevalent and associated with greater disease activity and functional impairment. At least moderate depression was found in 15% of patients, although estimates varied depending on the criteria and thresholds selected; pooled estimates of prevalence of depression in axSpA were 38 to 52% using lower HADS thresholds and the SDS.

The strength of this meta-analysis lies in its strict inclusion criteria, as prevalence estimates can vary significantly depending on variable sampling methods and definition for depression. The included studies had high quality overall and low risk of publication bias. However, overall heterogeneity of prevalence estimates remained high, largely due to the wide range of screening or diagnostic tools used to define depression. Heterogeneity was improved when estimates were pooled by depression definition, and further reduced in sensitivity analyses excluding the studies by Hakkou et al. and Jiang et al. These two studies highlight the importance of age, which was inversely associated with depression prevalence, and socioeconomic status, which future studies could approximate by grouping by continent or gross domestic product.

The main limitation of the meta-analysis of prevalence was that almost all studies used screening criteria to detect depression. This may lead to over-estimation of the prevalence of true depression. Using diagnostic criteria, Chan et al. found one of the lowest prevalences of depression. However, they reported a sensitivity and specificity of 82 and 79%, respectively, for HADS ≥ 8 in their axSpA cohort, using SCID as the gold standard [24]. Almost all studies described hospital cohorts which are likely to have more severe disease than a random or primary care sample. However, disease activity was not found to be associated with prevalence estimates; although the RCT cohort had much higher disease activity than observational cohorts, it reported similar prevalence of depression. The higher disease activity and functional impairment found in patients with depression were unadjusted for confounders such as smoking and deprivation. It is possible that adjusted effect sizes could be smaller, although data from our own axSpA cohort [10] found that doing so made little difference to the effect size of depression. Lastly, the direction of the causal relationship between depression and axSpA disease severity could only be speculated from these studies of cross-sectional association.

The prevalence of depression in axSpA was higher than that of the general population [37], but very similar to those reported in rheumatoid arthritis (RA) cohorts. A meta-analysis of RA patients reported depression in 15% using HADS ≥ 11 and 34% using HADS ≥ 8 [38]. This similarity was unexpected since the RA cohorts were mostly female. Given that depression is more prevalent in females in the general population [8], it is possible that more male axSpA patients were reporting depression than male RA patients. However, our finding that the prevalence of depression was similar between AS and nr-axSpA groups suggest that gender may not be as significant a risk factor for depression in axSpA.

Patients with depression had significantly worse disease activity and functional impairment across most indices. As an example, the pooled difference in BASDAI was 1.4 units, which is clinically relevant as ≥ 2-unit improvement is considered as response to TNF inhibitors (TNFi) [39]. The direction of this potential causal relationship cannot be inferred from studies included in this meta-analysis. Patients with more severe disease may be at higher risk of developing comorbid depression. Conversely, depression has been shown to exacerbate the perception of pain [40] and may cause axSpA patients with depression to report greater disease severity. This may explain why the differences in subjective indices (BASDAI, spVAS, BASFI) were larger than differences in objective measures (BASMI, ASDAS). It is interesting that the difference in BASDAI between groups with and without depression were similar regardless of severity of depression; the presence of any level of depression may adversely influence response to the BASDAI questionnaire.

In healthcare systems where TNFi can only be continued on the basis of demonstrable response, such as in the UK, efforts should be made to identify and manage comorbid depression to avoid withdrawing efficacious therapy inappropriately. More objective measures of disease activity, such as ASDAS, may be more robust to the influence of depression. Longitudinal axSpA studies are needed to evaluate the impact of depression on treatment outcomes; we have previously demonstrated, in a longitudinal RA cohort, that depression at baseline adversely affects treatment outcomes [41]. Equally, inflammation has been implicated in the pathophysiology of depression and may contribute to non-responsiveness to antidepressant therapies [9]. Longitudinal studies could also shed light on whether TNFi improves severity of depressive symptoms.

## Conclusions

Depression is common among axSpA patients and is associated with worse disease activity and functional impairment. Clinicians should be mindful of comorbid depression when managing axSpA patients, especially for younger patients and those with severe disease activity and functional impairment. Patients with depression should be appropriately referred and managed. This is especially pertinent if depressive symptoms are thought to adversely influence assessments of treatment response.

## Additional file

Additional file 1: **Tables S1**-**S3**, **Figures S1**-**S3.** (DOCX 71 kb)

## Abbreviations

ACR: American College of Rheumatology
AS: Ankylosing spondylitis
ASAS: Assessment of SpondyloArthritis international Society
ASDAS: Ankylosing Spondylitis Disease Activity Score
axSpA: Axial spondyloarthritis
BASDAI: Bath Ankylosing Spondylitis Disease Activity Index
BASFI: Bath Ankylosing Spondylitis Functional Index
BASMI: Bath Ankylosing Spondylitis Metrology Index
BSR: British Society for Rheumatology
CRP: C-reactive protein
EULAR: European League Against Rheumatism
HADS: Hospital Anxiety and Depression Scale depression subscale
LFK index: Luis Furuya-Kanamori index
PHQ-9: Patient Health Questionnaire
PROSPERO: International prospective register of systematic reviews
SCID: Structured Clinical Interview for Diagnostic and Statistical Manual of Mental Disorders
SDS: Zung Self-Rating Depression Scale
WMD: Weighted mean difference
