# Supplementary material for: The prevalence of depression in axial spondyloarthritis and its association with disease activity: a systematic review and meta-analysis
Source: Arthritis Res Ther. 2018 Jul 11;20:140. doi: 10.1186/s13075-018-1644-6 (PMC6042424; doi:10.1186/s13075-018-1644-6)
Supplement: Supplementary file 1 — Tables S1-S3, Figures S1-S3. (DOCX 71 kb) [file 13075_2018_1644_MOESM1_ESM.docx]

| Table S1. Health States Quality Index. |
| --- |
| 1.       Were the target population and the observation period well defined? |
| Yes = 1                No = 0 |
| 2.       Diagnostic criteria |
| Use of diagnostic system reported = 1                Own system/no system/not specified = 0 |
| 3.       Method of case ascertainment |
| Community survey/multiple institutions = 2                Inpatient/inpatients and outpatients/case registers = 1                Not specified = 0 |
| 4.       Administration of measurement protocol |
| Administered interview = 3                Systematic casenote review = 2                Chart diagnosis/case records = 1                Not specified = 0 |
| 5.       Catchment Area |
| Broadly representative (national or multi-site) = 2                Small area/not representative (single centre / university) = 1  Convenience sampling/ other (primary care / treatment group) = 0 |
| 6.       Prevalence measure |
| Point prevalence = 2                12-month prevalence = 1                Lifetime prevalence = 0 |

769 full-text articles (Pubmed 198, Web of Science 303, Medline 170, PsycINFO 32, Cochrane Library 1, CINAHL Plus 65)

40 conference abstracts (ACR 21, BSR 2, EULAR 17)

501 duplicates removed

308 titles and abstracts screened

30 did not specify use of AS/axSpA diagnostic criteria or specific cohort,

4 used the same population as already included studies,

20 did not specify validated depression criteria or threshold

16 studies included

(15 full-texts and 1 abstract)

70 studies assessed for full-text eligibility

238 studies irrelevant or did not report prevalence of depression

Figure S1. Flowchart of study selection.

| Table S2. Quality assessment using the 11-point Health States Quality Index. | | | | | | | | |
| --- | --- | --- | --- | --- | --- | --- | --- | --- |
| Study | Target population and observation period (0-1) | Diagnostic criteria (0-1) | case ascertainment (0-2) | Measurement protocol  (0-3) | Catchment Area (0-2) | Prevalence measure (0-2) | Total (Max: 11) | Quality weight |
| aAtes 2015 | 0 | 1 | 0 | 3 | 0 | 2 | 6 | 0.6 |
| Baysal 2011 | 1 | 0 | 1 | 3 | 2 | 2 | 9 | 0.9 |
| Chan 2014 | 1 | 1 | 1 | 3 | 1 | 2 | 9 | 0.9 |
| Dougados 2017 | 1 | 1 | 2 | 3 | 0 | 2 | 9 | 0.9 |
| Günaydin 2009 | 1 | 1 | 1 | 3 | 1 | 2 | 9 | 0.9 |
| Hakkou 2011 | 1 | 1 | 1 | 3 | 1 | 2 | 9 | 0.9 |
| Healey 2011 | 1 | 1 | 2 | 3 | 2 | 2 | 11 | 1.0 |
| Hyphantis 2013 | 1 | 1 | 1 | 3 | 1 | 2 | 9 | 0.9 |
| Jiang 2018 | 1 | 1 | 2 | 3 | 1 | 2 | 10 | 1.0 |
| Kilic 2014 | 1 | 1 | 1 | 3 | 1 | 2 | 9 | 0.9 |
| MacFarlane 2017 | 1 | 1 | 2 | 3 | 2 | 2 | 11 | 1.0 |
| Martindale 2006 | 1 | 1 | 1 | 3 | 1 | 2 | 9 | 0.9 |
| Rodríguez-Lozano 2012 | 1 | 1 | 1 | 3 | 1 | 2 | 9 | 0.9 |
| Xu 2016 | 1 | 0 | 1 | 3 | 1 | 2 | 8 | 0.8 |
| Zhang 2016 | 1 | 0 | 1 | 3 | 1 | 2 | 8 | 0.8 |
| Zou 2016 | 1 | 0 | 1 | 3 | 1 | 2 | 8 | 0.8 |

Figure S2. Funnel plot (top) and Doi plot (bottom) showing no significant publication bias.

Figure S3. Pooled prevalence of depression in AS cohorts, grouped by criteria and threshold.

| Table S3. Comparing markers of disease activity and functional impairment between groups with and without depression. | | | | | | | |
| --- | --- | --- | --- | --- | --- | --- | --- |
| Study | Variable | No Depression | | | Depression | | |
|  |  | Number of Patients | Mean | SD | Number of Patients | Mean | SD |
| Baysal 2011  HADS (≥7)  Qi: 0.9 | BASDAI | 147 | 3.29 | 2.24 | 96 | 5.37 | 2.46 |
|  | spVAS | 147 | 3.96 | 2.72 | 96 | 6.01 | 2.97 |
|  | BASFI | 147 | 3.08 | 3.24 | 96 | 5.06 | 2.76 |
|  | BASMI | 147 | 3.36 | 2.57 | 96 | 3.91 | 2.66 |
|  | HAQ | 147 | 0.88 | 1.06 | 96 | 1.26 | 1.02 |
|  | ESR | 147 | 18.95 | 20.63 | 96 | 29.30 | 27.85 |
| Kilic 2014  HADS (≥7)  Qi: 0.9 | BASDAI | 177 | 2.8 | 2.11 | 139 | 4.61 | 2.19 |
|  | spVAS | 177 | 3.84 | 4.80 | 139 | 4.90 | 2.55 |
|  | BASFI | 177 | 1.88 | 1.97 | 139 | 3.4 | 2.45 |
|  | BASMI | 177 | 2.09 | 1.79 | 139 | 2.54 | 1.93 |
|  | ASDAS-CRP | 177 | 2.47 | 1.01 | 139 | 2.96 | 0.97 |
|  | CRP | 177 | 15.82 | 22.61 | 139 | 13.99 | 19.20 |
|  | ESR | 177 | 20.01 | 21.18 | 139 | 22.36 | 18.77 |
| Hakkou 2011  HADS (≥8)  Qi: 0.9 | BASDAI | 49 | 3.43 | 2.54 | 61 | 5.22 | 2.44 |
|  | BASFI | 49 | 3.96 | 2.8 | 61 | 6.79 | 2.68 |
|  | BASMI | 49 | 4.14 | 3.42 | 61 | 5.54 | 3.63 |
| Martindale 2006  HADS (≥11)  Qi: 0.9 | BASDAI | 78 | 4.61 | 2.23 | 11 | 6.8 | 1.28 |
|  | BASFI | 78 | 4.14 | 2.58 | 11 | 6.8 | 1.46 |
|  | BASMI | 78 | 3.21 | 1.71 | 11 | 4.52 | 1.55 |
| Zhang 2016  SDS (≥51)  Qi: 0.8 | BASDAI | 203 | 3 | 1.9 | 111 | 4.6 | 2.1 |
|  | spVAS | 203 | 3.2 | 2.5 | 111 | 5.0 | 2.7 |
|  | BASFI | 203 | 1.2 | 1.8 | 111 | 2.5 | 2.5 |
|  | BASMI | 203 | 1.3 | 1.9 | 111 | 1.7 | 1.9 |
|  | CRP | 203 | 1.2 | 1.5 | 111 | 2.3 | 5.4 |
|  | ESR | 203 | 14.6 | 16.8 | 111 | 20.8 | 21.5 |
| Zou 2016  SDS (≥53)  Qi: 0.8 | BASDAI | 34 | 2.58 | 1.55 | 26 | 4.44 | 1.82 |
|  | spVAS | 34 | 3.76 | 1.92 | 26 | 4.42 | 1.90 |
|  | BASFI | 34 | 1.36 | 1.78 | 26 | 2.79 | 1.97 |
|  | ASDAS-CRP | 34 | 2.59 | 0.96 | 26 | 3.07 | 0.98 |
|  | ASDAS-ESR | 34 | 2.29 | 0.87 | 26 | 2.73 | 0.93 |
|  | CRP | 34 | 20.16 | 21.02 | 26 | 17.42 | 15.68 |
|  | ESR | 34 | 23.62 | 22.44 | 26 | 20.65 | 16.19 |
| Xu 2016  SDS (≥53)  Qi: 0.8 | BASDAI | 65 | 1.89 | 1.7 | 38 | 3.53 | 2.53 |
|  | spVAS | 65 | 1.95 | 1.80 | 38 | 3.24 | 2.24 |
|  | BASFI | 65 | 0.36 | 0.89 | 38 | 1.27 | 1.65 |
|  | BASMI | 65 | 1.95 | 1.77 | 38 | 2.55 | 1.96 |
|  | HAQ | 65 | 1.14 | 3.38 | 38 | 2.85 | 4.49 |
|  | CRP | 65 | 12.3 | 8.3 | 38 | 16.4 | 9.1 |
|  | ESR | 65 | 16.3 | 15.1 | 38 | 19.7 | 17.6 |
| HADS, Hospital Anxiety and Depression Scale – depression subscale; SDS, Zung self-rating depression scale; BASDAI, Bath AS disease activity index; spVAS, spinal pain visual analogue scale; BASFI, Bath AS functional index; BASMI, Bath AS metrology index; HAQ, Health assessment questionnaire; ASDAS, AS disease activity score. | | | | | | | |
